# Network Pharmacology and Comparative Transcriptome Reveals Biotargets and Mechanisms of Curcumol Treating Lung Adenocarcinoma Patients With COVID-19

**DOI:** 10.3389/fnut.2022.870370

**Published:** 2022-04-19

**Authors:** Lu Yang, Hao Xiong, Xin Li, Yu Li, Huanhuan Zhou, Xiao Lin, Ting Fung Chan, Rong Li, Keng Po Lai, Xu Chen

**Affiliations:** ^1^Laboratory of Environmental Pollution and Integrative Omics, Guilin Medical University, Guilin, China; ^2^Guilin Center for Disease Control and Prevention, Guilin, China; ^3^Guangxi Key Laboratory of Tumor Immunology and Microenvironmental Regulation, Guilin Medical University, Guilin, China; ^4^Department of Psychiatry, Icahn School of Medicine at Mount Sinai, New York, NY, United States; ^5^State Key Laboratory of Agrobiotechnology, School of Life Sciences, The Chinese University of Hong Kong, Shatin, Hong Kong SAR, China; ^6^Department of Pharmacy, Guilin Medical University, Guilin, China

**Keywords:** lung adenocarcinoma, COVID-19, curcumol, network pharmacology, transcriptome

## Abstract

The coronavirus disease 2019 (COVID-19) pandemic has led to 4,255,892 deaths worldwide. Although COVID-19 vaccines are available, mutant forms of severe acute respiratory syndrome coronavirus 2 (SARS-CoV-2) have reduced the effectiveness of vaccines. Patients with cancer are more vulnerable to COVID-19 than patients without cancer. Identification of new drugs to treat COVID-19 could reduce mortality rate, and traditional Chinese Medicine(TCM) has shown potential in COVID-19 treatment. In this study, we focused on lung adenocarcinoma (LUAD) patients with COVID-19. We aimed to investigate the use of curcumol, a TCM, to treat LUAD patients with COVID-19, using network pharmacology and systematic bioinformatics analysis. The results showed that LUAD and patients with COVID-19 share a cluster of common deregulated targets. The network pharmacology analysis identified seven core targets (namely, AURKA, CDK1, CCNB1, CCNB2, CCNE1, CCNE2, and TTK) of curcumol in patients with COVID-19 and LUAD. Clinicopathological analysis of these targets demonstrated that the expression of these targets is associated with poor patient survival rates. The bioinformatics analysis further highlighted the involvement of this target cluster in DNA damage response, chromosome stability, and pathogenesis of LUAD. More importantly, these targets influence cell-signaling associated with the Warburg effect, which supports SARS-CoV-2 replication and inflammatory response. Comparative transcriptomic analysis on *in vitro* LUAD cell further validated the effect of curcumol for treating LUAD through the control of cell cycle and DNA damage response. This study supports the earlier findings that curcumol is a potential treatment for patients with LUAD and COVID-19.

## Key points

- COVID-19-/LUAD-associated and curcumol targets were identified.
- Prognostic value of curcumol against LUAD and COVID-19 was characterized.
- We identified seven core pharmacological targets of curcumol, namely, AURKA, CDK1, CCNB1, CCNB2, CCNE1, CCNE2, and TTK, in treating LUAD and COVID-19.
- Comparative transcriptomic analysis specified the effects of curcumol for treating LUAD through control of cell cycle, DNA damage response, and cell apoptosis.

## Introduction

Coronavirus disease 2019 (COVID-19) is a novel disease characterized by high infectivity and rapid spread. Widespread community transmission of severe acute respiratory syndrome coronavirus 2 (SARS-CoV-2) has led to a global pandemic. As of 4 March 2022, 440,807,756 confirmed cases of COVID-19 and 5,978,096 deaths had been reported worldwide, according to the World Health Organization (WHO) (https://worldhealthorg.shinyapps.io/covid/). A considerable proportion of patients with COVID-19 related critical illness have comorbidities, which are associated with increased mortality.

The prospective cohort studies have demonstrated that patients with COVID-19 with underlying malignancies have a higher mortality rate than those without cancer (1, 2). The recent studies have shown that the levels of angiotensin-I-converting enzyme 2 (ACE2) and transmembrane serine protease 2 (TMPRSS2) in patients with lung adenocarcinoma (LUAD) are significantly increased, and the increased levels of these enzymes are associated with susceptibility of patients with LUAD to SARS-CoV-2 (3), because ACE2 serves as an important binding site for SARS-CoV-2, leading to facilitate viral entry into target host cells (4). Lung adenocarcinoma is the most common subtype of lung cancer. The development of LUAD is stepwise, beginning with atypical adenomatous hyperplasia (AAH), and progressing to adenocarcinoma *in situ* (AIS) and then to minimally invasive adenocarcinoma (MIA) (5, 6). Most of the patients are diagnosed with advanced disease and have poor prognosis. Gene mutations (such as EGFR, KRAS, and BRAF mutations) and tumor inflammatory microenvironment are strongly associated with LUAD pathogenesis (7, 8).

A meta-analysis of patients with lung cancer and COVID-19, which included 13 studies, showed that the pooled mortality of patients with lung cancer and COVID-19 (up to 25–42%) was significantly higher than the mortality of patients with other cancers (9, 10). This may be due to different pathophysiological factors, such as pulmonary compromise and smoking history, in patients with lung cancer, compared with other cancers (11). Therefore, there is an urgent need to identify drugs to treat patients with lung cancer and COVID-19. In addition to helping these patients, identification of such drugs will also relieve the pressure on respiratory healthcare services.

The current research findings have shown that traditional Chinese medicine (TCM), such as *Lianhua Qingwen Keli*, Honeysuckle Flower Cold-Relieving Granules, and *Xuebijing* injection can be effective in preventing COVID-19 and relieving clinical symptoms of COVID-19. These TCM were officially recommended by the National Medical Products Administration, as adjunctive therapy, in the treatment of COVID-19 (12). TCM can potentially be used for the treatment of comorbidities associated with COVID-19, and has received worldwide attention. Curcumol, a sesquiterpenoid isolated from Curcumae rhizoma, has been shown to have various therapeutic effects, including anticancer, antioxidant, antimicrobial, and anti-inflammatory effects (13). An *in vitro* study by Li et al. showed that curcumol suppresses proliferation of the LUAD cells, A549 and H460, by arresting the cell cycle, altering the expression of apoptosis signaling pathways and inducing tumor cell apoptosis (14). In chronic asthmatic mice, curcumol was found to reduce pulmonary inflammation and airway remodeling by decreasing cytokine levels (15). In addition, curcumol was reported to inhibit LUAD growth and metastasis and overcome tumor necrosis factor-related apoptosis-inducing ligand (TRAIL) resistance in lung cancer (16). The results of the aforementioned studies show that curcumol may be a potential treatment for patients with LUAD and COVID-19.

In the current study, we used network pharmacology, comparative transcriptome, and systematic bioinformatics analysis to investigate the use of curcumol for COVID-19 and LUAD treatment. We aimed to identify possible therapeutic targets and to unfold the molecular mechanisms underlying the therapeutic effects of curcumol in COVID-19 and LUAD, using clinicopathological analysis, gene ontology, KEGG enrichment analysis, ingenuity pathway analysis (IPA) and molecular docking.

## Materials and Methods

### Identification of Common Deregulated Targets Between COVID-19 and LUAD

For the identification of COVID-19-associated targets, the keywords “coronavirus COVID-19,” “coronavirus Disease 2019,” “severe acute respiratory syndrome coronavirus 2,” and “COVID-19” were subjected to different databases, including the Genecards database (17), Online Mendelian Inheritance in Man (OMIM) database (https://omim.org/), Therapeutic Target Database (TTD) (18), Comparative Toxicogenomics Database (CTD) (19), and National Center for Biotechnology Information (NCBI) (https://www.ncbi.nlm.nih.gov/), genes with the relevance score >1 were obtained from the databases. To identify LUAD-associated targets, the transcriptome data of patients with LUAD were obtained from The Cancer Genome Atlas (TCGA) database (https://portal.gdc.cancer.gov/) on 26 July 2021. Using the *limma* package in R on Bioconductor software, genes with FDR < 0.05, and |logfold change| > 2 were considered as differentially expressed genes (DEGs) (20).

### Identification of Pharmacological Targets of Curcumol in LUAD and COVID-19 Treatment

The pharmacological targets of curcumol were determined using various online tools and databases, including Swiss Target Prediction database and Bioinformatics Analysis Tool for Molecular mechANism of TCM (BATMAN-TCM) (21). The target genes were subjected to UniProt for human database correction. The common deregulated genes between COVID-19 and LUAD, that were previously identified, were intersected and compared with the targets of curcumol. Interactions between common targets were analyzed using the STRING database (version 11.0) (22) and Cytoscape software (version 3.6.1) (23). The Database for Annotation, Visualization and Integrated Discovery (DAVID) v6.8 was used for the gene ontology (GO) function enrichment analysis and KEGG enrichment analysis, to understand the functional roles of targets and signaling pathways controlled by the genes.

### Binding of Curcumol to Predicted Targets

Molecular docking analysis was used to investigate the possible binding between curcumol and its predicted targets. The protein structures of the core targets were searched for from the Protein Data Bank (PDB) database (24). Identified protein structures were then docked with curcumol using the AutoDock Vina program and docking analysis was conducted (25).

### The Roles of Curcumol Target Genes in LUAD Pathogenesis

To determine the pathological roles of the core targets in LUAD, Cox proportional hazards models were applied in univariate analysis of survival as a function of clinical variables and gene expression.

### Cell Culture

The human lung adenocarcinomic cell lines of A549 were incubated with high glucose dulbecco's modified eagle medium (DMEM) medium (ThermoFisher, Cat. No. 11965118), supplemented with 0.5% penicillin–streptomycin (ThermoFisher, Cat. No. 15140122) and 5% fetal bovine serum (ThermoFisher, Cat. No. 10082147) under 5% CO_2_ at 37°C.

### Cell Proliferation Assays

The cells were seeded in a 96-well plate at a cell density of 2 × 10^4^ cells per well, with eight replicate wells. The cells were treated with different concentrations of curcumol (0.1–100 μM) for 48 h. After the incubation, the cell viability was measured by the CCK-8 assay (Data Inventory Biotechnology) as described previously (26). The colorimetric product formed was measured at an absorbance of 450 nm and 600 nm, ΔOD = OD_450nm_ – OD_600nm_.

### The RNA Sequencing

After the treatment of the cell with 100 μM curcumol for 48 h, the total RNA of the cell was extracted using Trizol reagent (Thermofisher) following the manufacturer's instruction. The RNA quality and quantity were assessed by using Bioanalyzer 2,100 and RNA 6,000 Nano LabChip Kit (Agilent), high-quality RNA samples with RNA integrity number (RIN) number higher than 7.0 were used to construct sequencing library. The average insert size for the final complementary DNA (Cdna) library was about 300 bp. Then 2 × 150 bp paired-end sequencing (PE150) was performed on an Illumina Novaseq™. The high-quality clean reads were mapped to the Human genome reference (Homo sapiens Ensembl v96) using HISAT2 software (version: hisat2-2.0.4) (27). StringTie and ballgown were used to determine the gene expression level (28). The genes with a 1.5 < fold change (treatment/control) < 0.75 and –log10 (*q*-value) > 1.3 were considered as DEGs. The DEGs were subjected to the DAVID v6.8 analysis (29) and IPA (https://www.qiagenbioinformatics.com/products/ingenuity-pathway-analysis) to delineate the molecular mechanism underlying the effect of curcumol for treating LUAD.

## Results

### Identification of Pharmacological Targets of Curcumol in COVID-19 and LUAD Treatment

**Figure 1:**
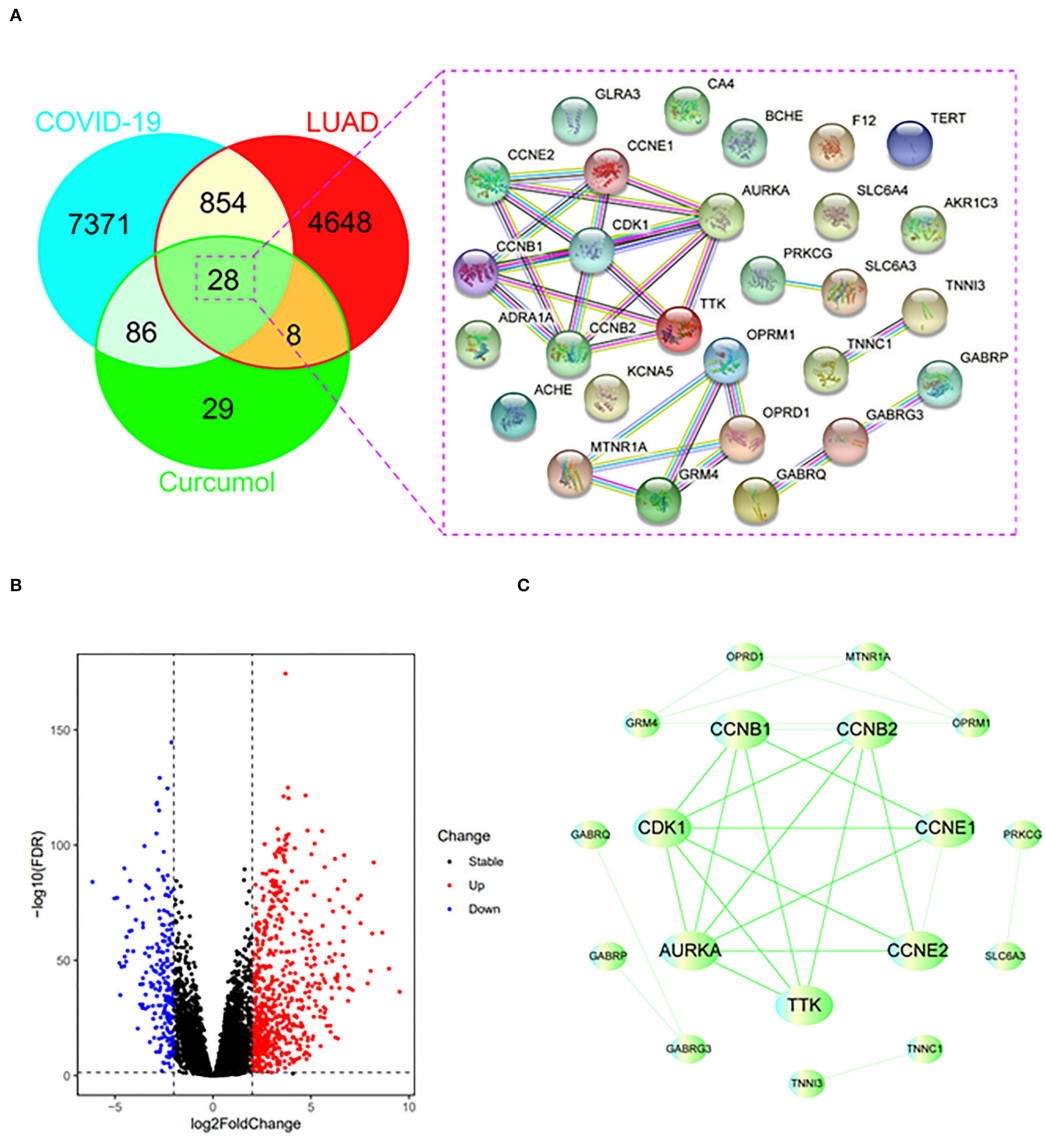
Identification of curcumol-targeted COVID-19- LUAD-associated targets. **(A)** Venn diagram showing the intersecting targets of curcumol /COVID-19/LUAD. **(B)** Volcano plot showing the expression level of differential expressed COVID-19- LUAD-associated genes in patients with LUAD. The genes with |log_2_ (fold change)| > 1 and –log10(FDR) > 1.3 were considered as differential expressed genes. **(C)** Protein–protein interaction analysis of curcumol/COVID-19/LUAD-intersecting genes using STRING tool.

Using the relevant databases, we identified a total of 8,339 targets associated with COVID-19 (Figure 1A). Using the TCGA database, we found 5,538 differential expressed genes associated with LUAD (Figure 1A). When we compared the COVID-19- and LUAD-associated targets, we found 882 shared targets (Figure 1A), among which 216 were downregulated and 666 were upregulated, in patients with LUAD (Figure 1B). To understand the pharmacology of curcumol, a network pharmacology analysis was conducted. We identified 151 curcumol-associated targets using the mentioned databases (Figure 1A) and, after comparison of the curcumol-associated targets with the COVID-19/ LUAD-associated targets, we found 28 targets shared by curcumol, COVID-19, and LUAD (Figure 1A). The molecular network analysis using Cytoscape highlighted seven core targets of curcumol, namely, AURKA, CDK1, CCNB1, CCNB2, CCNE1, CCNE2, and TTK in COVID-19 and LUAD (Figure 1C and Table 1).

**Table 1:** All seven core genes of curcumol against COVID-19 and LUAD.

**Protein Name**	**Symbol**	**Uniprot ID**
Aurora kinase A	AURKA	O14965
Cyclin-dependent kinase 1	CDK1	P06493
G2/mitotic-specific cyclin-B1	CCNB1	P14635
G2/mitotic-specific cyclin-B2	CCNB2	O95067
G1/S-specific cyclin-E1	CCNE1	P24864
G1/S-specific cyclin-E2	CCNE2	O96020
Dual specificity protein kinase TTK	TTK	P33981

### Binding of Curcumol to Targets CDK1, TTK, and AURKA

**Figure 2:**
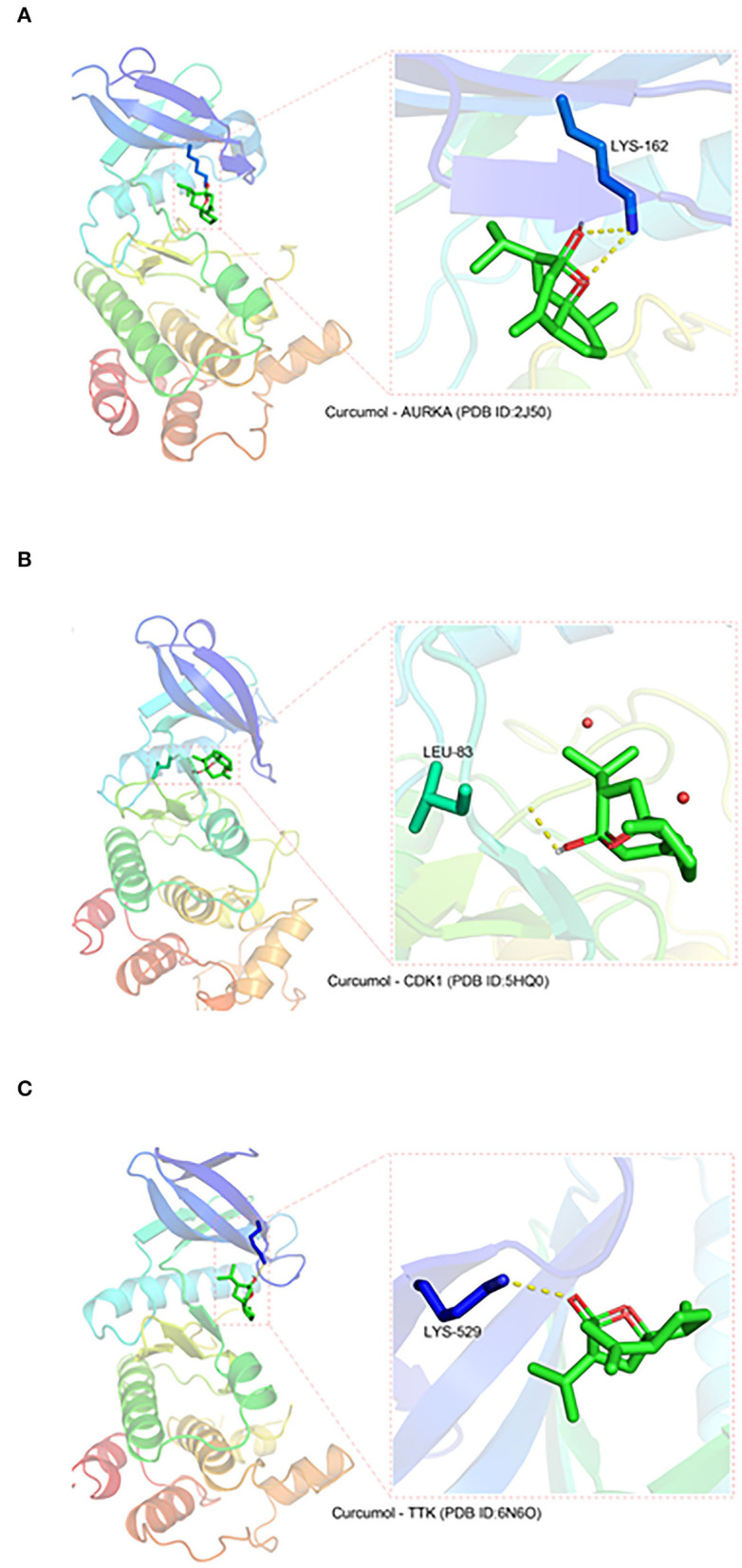
The direct binding of curcumol to CDK1, TTK, and AURKA. The protein structures of CDK1, TTK, and AURKA, obtained from the PDB database. Using the programs Autodock and Visual Molecular Dynamics for visualization, hydrogen bonds can be seen between curcumol and **(A)** the amino acid residue LYS-162 (3.2 Å) of AURKA (PDB ID: 2J50), **(B)** the amino acid residue LEU-83 (2.2 Å) of CDK1 (PDB ID: 5HQ0), and **(C)** the amino acid residue LYS-529 (3.0 Å) of TTK (PDB ID: ID:6N6O).

Three protein structures, CDK1, TTK, and AURKA, out of the seven core targets were available on the PDB database (AURKA, ID:2J50; CDK1, ID:5HQ0, and TTK, ID:6N6O). These protein structures were subjected to docking analysis with curcumol using the AutoDock Vina program. The results were displayed using PyMOL (version 2.3), which showed that curcumol formed a hydrogen bond with LYS-162 (3.2 Å) of AURKA (PDB ID: 2J50) (Figure 2A), and the binding affinity of curcumol for AURKA was −6.4 kcal/mol. A similar bindings were observed between curcumol and the amino acid residue LEU-83 (2.2 Å) of CDK1 (PDB ID: 5HQ0) (Figure 2B) and between curcumol and the amino acid residue LYS-529 (3.0 Å) of TTK (PDB ID: ID:6N6O) (Figure 2C). The binding affinities of curcumol for CDK1 and TTK were −3.2 kcal/mol and −4.7 kcal/mol, respectively. Our data suggested the direct binding of curcumol to its targets.

### The Roles of Curcumol Target Genes in LUAD Pathogenesis

**Figure 3:**
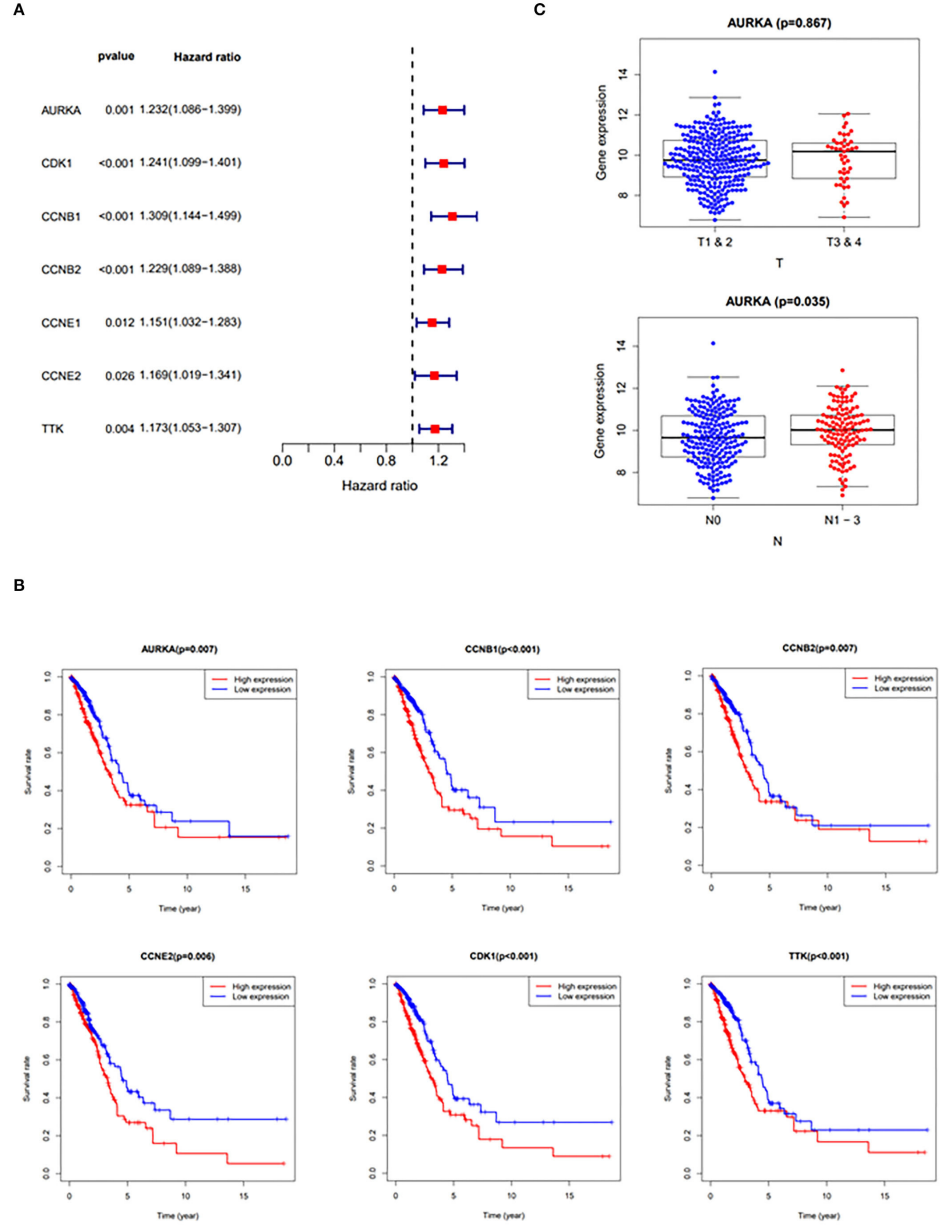
Clinicopathologic analysis of curcumol-targeted COVID-19/LUAD-associated targets. **(A)** The Univariate Cox proportional hazards models showing that the expression of curcumol target genes in COVID-19 and LUAD was significantly associated with relative risk of survival in the patients with LUAD. **(B)** The survival analysis using Kaplan–Meier estimator showing that the patients with LUAD with higher expression of AURKA, CDK1, CCNB1, CCNB2, CCNE2, and TTK had poorer overall survival rates. **(C)** The higher expressions of AURKA in patients with LUAD are associated with advanced stages of LUAD. T, Staging of tumor; N, Number of lymph nodes containing tumor.

The results of the hazards models showed that the expression of curcumol targets in COVID-19 and LUAD was significantly associated with the relative risk of survival [AURKA (*p* = 0.001, hazard ratio, 1.086–1.399); CDK1 (*p* < 0.001, hazard ratio, 1.099–1.401); CCNB1 (*p* < 0.001, hazard ratio, 1.144–1.499); CCNB2 (*p* < 0.001, hazard ratio, 1.089–1.388); CCNE1 (*p* = 0.012, hazard ratio, 1.032–1.283); CCNE2 (*p* = 0.026, hazard ratio, 1.019–1.341); TTK (*p* = 0.004, hazard ratio, 1.053–1.307)] in patients with LUAD (Figure 3A). The results of survival analysis using the Kaplan–Meier estimator also showed that patients with LUAD with greater expression of the genes, AURKA, CDK1, CCNB1, CCNB2, CCNE2, and TTK, had poorer overall survival rates (Figure 3B). In addition, the correlation analysis highlighted that increased expression of AURKA was associated with advanced stages of LUAD and the increased number of lymph nodes containing tumor (Figure 3C).

### Curcumol Target Genes Mediated DNA Damage Response and Cell Cycle Control

**Figure 4:**
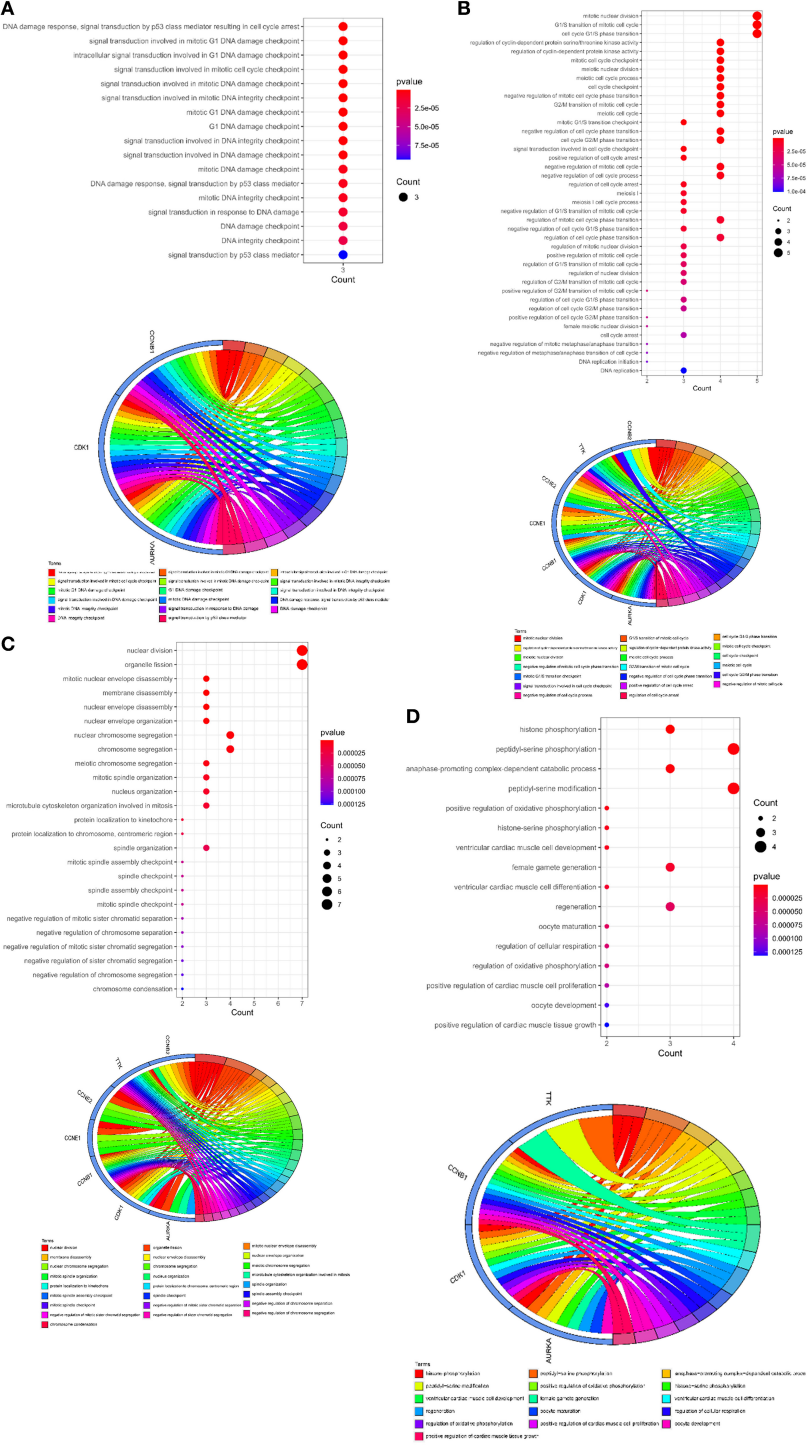
Functional characterization of curcumol-targeted COVID-19/ LUAD-associated targets. **(A)** The GO enrichment analysis highlighted the biological processes related to DNA damage response that are controlled by curcumol-targeted COVID-19/LUAD-associated targets. The circos plot showing the involvement of AUKRA, CDK1, and CCNB1 in the enriched biological processes. **(B)** The GO enrichment analysis highlighted the biological processes related to cell cycle control regulated by curcumol-targeted COVID-19/LUAD-associated targets. Circos plot showing the involvement of AUKRA, CDK1, CCNB1, CCNB2, CCNE1, CCNE2, and TTK in the enriched biological processes. **(C)** The GO enrichment analysis highlighted the biological processes related to cellular assembly processes controlled by curcumol targeted COVID-19/LUAD-associated targets. Circos plot showing the involvement of AUKRA, CDK1, CCNB1, CCNB2, CCNE1, CCNE2, and TTK in the enriched biological processes. **(D)** The GO enrichment analysis highlighted the biological processes related to carcinogenesis of LUAD. Circos plot showing the involvement of AUKRA, CDK1, CCNB1, and TTK in the enriched biological processes. The size of the dot represents the number of targets. The color intensity of the dot represents the significance of the processes.

To further understand the biological roles of curcumol in the treatment of COVID-19 and LUAD, the identified core targets were subjected to GO enrichment analysis and KEGG pathway enrichment analysis. The results of GO analysis showed that curcumol targets (AURKA, CDK1, and CCNB1) could mediate biological processes related to DNA damage response by controlling DNA integrity and the DNA damage checkpoint (Figure 4A and Supplementary Table 1), or by regulating cyclin-dependent protein kinase activity, which could lead to cell cycle arrest at different cell cycle checkpoints, such as cell cycle G1/S phase transition and cell cycle G2/M phase transition (Figure 4B and Supplementary Table 1). These responses are controlled by the seven curcumol targets (Figure 4B and Supplementary Table 1). The other possible outcome of mediation of biological processes related to the DNA damage response by these targets is the alteration of cellular assembly processes, such as organelle fission, mitotic nuclear envelope disassembly, chromosome segregation, and mitotic spindle organization (Figure 4C and Supplementary Table 1). In addition, curcumol targets were found to regulate many important processes involved in LUAD carcinogenesis, such as histone phosphorylation, oxidative phosphorylation, and cellular respiration (Figure 4D and Supplementary Table 1).

**Figure 5:**
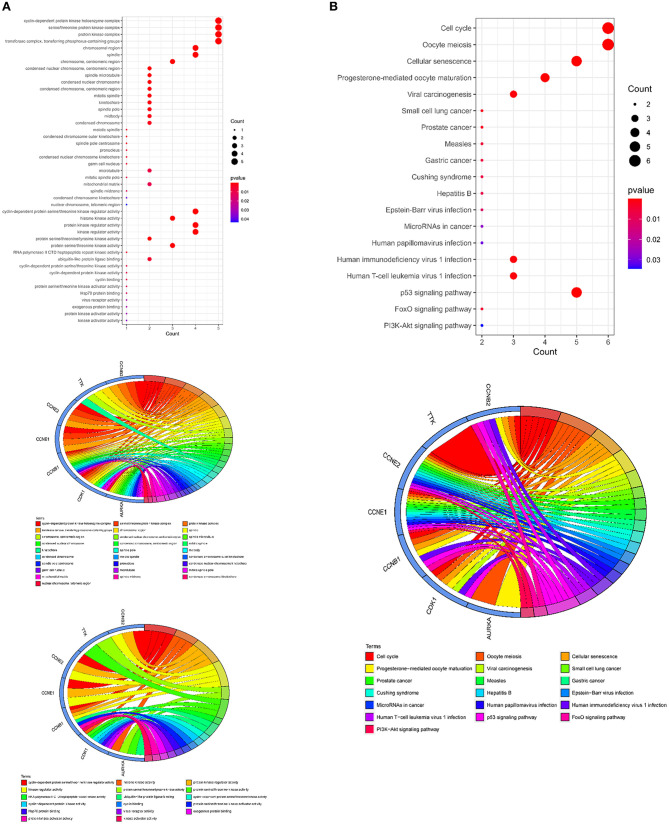
Molecular functions and signaling pathways controlled by curcumol-targeted COVID-19/LUAD-associated targets. **(A)** The GO enrichment analysis highlighted the involved enzymatic complexes, chromosome compartments, and molecular functions in curcumol-targeted COVID-19/LUAD-associated targets. The size of the dot represents the number of targets. The color intensity of the dot represents the significance of the terms. The Circos plot shows the involvement of AUKRA, CDK1, CCNB1, CCNB2, CCNE1, CCNE2, and TTK in the enriched terms. **(B)** The KEGG analysis highlighted the cell-signaling pathways related to cancer development, mediated by curcumol, against COVID-19 and LUAD. The size of dot represents the number of targets. The color intensity of the dot represents the significance of the pathways. The Circos plot shows the involvement of AUKRA, CDK1, CCNB1, CCNB2, CCNE1, CCNE2, and TTK in the enriched signaling.

The GO cellular components and molecular functions analyses showed that the curcumol targets play roles in many enzymatic complexes related to cell cycle control, such as in cyclin-dependent protein kinase holoenzyme complex and in serine/threonine protein kinase complex (Figure 5A and Supplementary Table 2). More importantly, the targets are involved in chromosome organization, specifically, in organization of centromeric region of chromosomes, kinetochores, mitotic spindle pores, and telomeric regions (Figure 5A and Supplementary Table 2). The results of the molecular function analysis further highlighted the effects of curcumol on cyclin-dependent protein serine/threonine kinase regulator activity, histone kinase activity, and cyclin binding (Figure 5A and Supplementary Table 2). KEGG pathway analysis was used to investigate the pharmacological effect of curcumol on the regulation of cell-signaling pathways. The results showed that curcumol could target many cell-signaling pathways related to cancer development (Figure 5B and Supplementary Table 3), including the p53 signaling pathway, FoxO signaling pathway, and PI3K–Akt signaling pathway (Figure 5B and Supplementary Table 3). The curcumol targets were also found to play a role in immune response to viral infections, like T-cell leukemia virus 1 infection, papillomavirus infection, and immunodeficiency virus 1 infection (Figure 5B and Supplementary Table 3).

### Curcumol Inhibited the Cell Proliferation of LUAD Through the Control of Cell Cycle and DNA Damage

**Figure 6:**
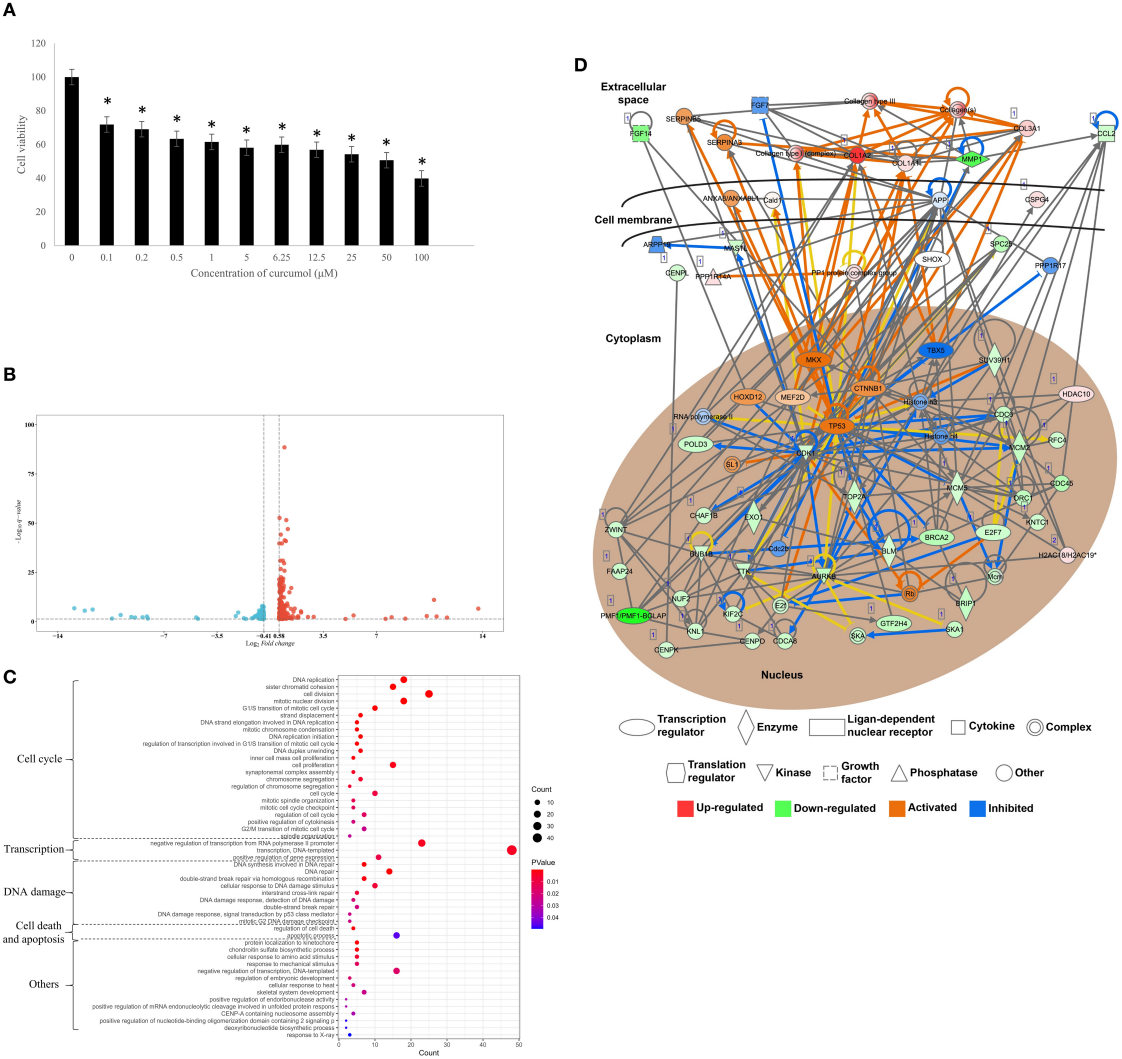
Curcumol-targeted genes involved in cell cycle and DNA damage of LUAD cell model A549. **(A)** The curcumol treatment (100 μM) inhibited cell proliferation of LUAD cell in a dose-dependent manner; *N* = 8 and three experiments. **(B)** The comparative transcriptomic analysis showed the differential gene expression in LUAD cell caused by curcumol treatment (100 μM). Genes with a 1.5 < fold change (treatment/control) < 0.75 and –log10 (*q*-value) > 1.3 were considered as DEGs. Blue dots represented downregulated genes and red dots represented upregulated genes. **(C)** The GO enrichment analysis highlighted the importance of curcumol-dysregulated genes in cell cycle, DNA damage, and cell apoptosis of LUAD cell. The size of bubble represented the number of DEGs involved in the processes, the color of bubble represented the significance of the processes. **(D)** Gene networking of IPA highlighted the involvement of DEGs in cell cycle and DNA damage response. Red color represented the upregulated genes, green color represented downregulated genes. Orange represented the predicted activated molecules and blue color represented the predicted inhibited molecules in the signaling. ^*^ represented *p* < 0.05, as compared to control group.

To investigate the effect of curcumol on lung cancer, an *in vitro* LUAD model A549 was used. Our result showed that the treatment of curcumol caused a significant dose-dependent inhibition of cell proliferation in LUAD as compared to the control group (Figure 6A). Then comparative transcriptomic analysis was conducted to delineate the corresponding molecular mechanism. By comparing the gene expression profile of control and curcunol treatment group, we found 348 DEGs, including 206 upregulated genes and 142 downregulated genes (Figure 6B and Supplementary Table 4). The DEGs were used for the DAVID and IPA analysis to understand the biological alteration and gene network mediated by curcumol treatment in LUAD. The result of GO enrichment showed that the curcumol treatment controlled biological processes related to cell cycle and gene transcription of LUAD (Figure 6C and Supplementary Table 5). More importantly, curcumol could trigger DNA damage response, leading to cell death and cell apoptosis in LUAD (Figure 6C and Supplementary Table 5). These results further supported the above findings from network pharmacology. Finally, gene networking of IPA highlighted the involvement of transcription and translation factors, enzymes, kinases, phosphatases, and receptors in curcumol-mediated cell cycle and DNA response (Figure 6D and Table 2).

**Table 2:** Highlighted IPA canonical pathways.

**Ingenuity canonical pathways**	**–log (*p*-value)**	**Gene symbol**
Kinetochore metaphase signaling pathway	1.61E+01	AURKB,BUB1B,CDCA8,CDK1,CENPK,CENPL,CENPO,H2AC18/H2AC19,KIF2C,KNL1, KNTC1,MASTL,NUF2,PMF1/PMF1-BGLAP,PPP1R14A,SKA1,SPC25, STAG3,TTK,ZWINT
Cell cycle control of chromosomal replication	4.89E+00	CDC45,CDC6,CDK1,MCM2,MCM5,ORC1,TOP2A
Role of BRCA1 in DNA damage response	3.04E+00	BLM,BRCA2,BRIP1,E2F7,FAAP24,RFC4
Tumor microenvironment pathway	3.00E+00	CCL2,COL1A1,COL1A2,COL3A1,CSPG4,FGF14,MMP1, MMP17,MMP28
NER (Nucleotide Excision Repair, Enhanced Pathway)	1.82E+00	CHAF1B,GTF2H4,POLD3,RFC4,TOP2A
Mismatch repair in eukaryotes	1.68E+00	EXO1,RFC4
Cyclins and cell cycle regulation	1.51E+00	CDK1,E2F7,HDAC10,SUV39H1
Role of CHK proteins in cell cycle checkpoint control	1.34E+00	CDK1,E2F7,RFC4

## Discussion

Using the network pharmacology analysis, we identified seven core targets of curcumol against COVID-19 and LUAD. These seven targets, namely, CCNB1, CCNB2, CCNE1, CCNE2, AURKA, CDK1, and TTK, have been reported to play critical roles in the carcinogenesis and development of LUAD. These targets mediate signaling pathways, such as PI3K/AKT and p53, that are associated with the Warburg effect, which supports SARS-CoV-2 replication and inflammatory response (30, 31). Four cyclin family members (CCNB1, CCNB2, CCNE1, and CCNE2) were identified as core targets in our analysis. Also, cyclin B1 (CCNB1) is associated with poor prognosis in LUAD (32). It is a downstream effector of monoacylglycerol lipase (MGLL), a key enzyme in lipid metabolism which plays an oncogenic role in LUAD progression and metastasis (33). The cyclin family member CCNB1 also contributes to lung inflammation and oxidative stress (34, 35); CCNB2 is an independent predictor of the prognosis of patients with LUAD (36). The functional characterization further highlighted the involvement of CCNB2 in inducing cell cycle arrest and apoptosis in LUAD cells (37). Moreover, the high levels of CCNB2 activate inflammation-induced motility in LUAD (38). Additionally, a study by Ma et al. revealed that knockdown of CCNB2 suppressed proliferation of the LUAD cell line (39). In addition to cyclin B members, our results show that cyclin E members, such as CCNE1 and CCNE2, are also targeted by curcumol; CCNE1 plays a role in progression, cell proliferation, and cell cycle arrest of lung cancer cells (40–42).

Using molecular docking, we found that curcumol binds directly to a group of kinases, including CDK1, TTK, and Aurora kinase A (AURKA). AURKA, a cell cycle kinase, is associated with many cancer types (43). An *in vitro* study of human LUAD cell lines demonstrated that AURKA plays an important role in the proliferation of LUAD cells, through the regulation of multiple downstream effectors, such as RAF-1, CCND2, CCND3, CDK4, PAK4, and EGFR (44). In addition to its function as a cell cycle kinase, AURKA is considered as an inhibitor which prevents the chromatin assembly of functional replisomes, leading to sensitization of cancer cells to combination therapy (45). Further functional characterization studies of AURKA report that AURKA suppression enhances the radiosensitivity of lung cancer and its response to EGFR inhibitors (46, 47). In addition, the downregulating AURKA inhibits docetaxel chemoresistance in LUAD (48), suggesting that AURKA is a promising target for LUAD therapy. Many studies have also demonstrated the autoimmune and inflammatory roles of AURKA *via* regulation of M1 macrophage polarization (49, 50).

Our results showed that the curcumol targets the other cell cycle gatekeeper kinases, such as cyclin-dependent kinase 1 (CDK1); CDK1 is a potential prognostic biomarker of/and target for lung cancer (51); CDK1 activity is critical for JAK/STAT3 signaling activation, and the inhibition of CDK1 can suppress lung cancer (52). In addition, an *in vitro* study of LUAD cells showed that reduced CDK1 activity led to cell cycle arrest and promotion of apoptosis in LUAD (53, 54). CDK1 controls many effectors involved in cell cycle regulation, such as FOXM1, TRAP1, and GCN1 (55–57). Furthermore, CDK1 plays a role in the DNA damage response. For example, CDK1 ensures optimal Fun30 phosphorylation and checkpoint activation at DNA double-strand breaks and plays an important role in the DNA damage response by preventing the formation of lagging chromosomes (58, 59). Also, CDK1 ensures accurate chromosomal segregation *via* the activity of acetyltransferase TIP60 and chromatin remodeller RSF1 (60, 61). Cumulative studies have reported that DNA damage is associated with higher mortality in patients with COVID-19 (62). Kinase threonine tyrosine kinase (TTK) is a critical component of the spindle assembly checkpoint (63). Also, TTK is a biomarker for prognosis of Non-small cell lung cancer (64), and the upregulation of TTK increases the cancer progression in lung cancer (65). In addition, the TTK antagonism has marked antineoplastic effects against LUAD (66). The targeting of CDK1 and TTK by curcomol provides an opportunity to treat patients with LUAD and COVID-19. The results were further validated by using comparative transcriptomic analysis on LUAD cell. The treatment of curcumol could inhibited cell proliferation of LUAD through its control on cell cycle and DNA damage response.

## Conclusions

In conclusion, we identified the pharmacological targets and the therapeutic mechanisms of curcumol in the treatment of COVID-19 and LUAD, including immune response, DNA damage response, and cell cycle arrest, and regulation of cell-signaling pathways such as the p53 signaling pathway, FoxO signaling pathway, and PI3K-Akt signaling pathway. The results were further supported by the comparative transcriptomic analysis on *in vitro* LUAD cell, suggesting that curcumol has potential for treating patients with LUAD and COVID-19. However, further Pre-clinical study is needed to warrant the findings of the present study before the clinical use.

## Data Availability Statement

Sequencing data of transcriptome sequencing that support the findings of this study have been deposited in the NCBI BioProject database (https://www.ncbi.nlm.nih.gov/bioproject) with the BioProject accession code PRJNA793079.

## Author Contributions

RL, KL, and XC contributed to the conception, design of the manuscript, drafted this manuscript, and revised this manuscript. LY, HX, XLi, YL, HZ, XLin, and TC contributed to the acquisition, analysis, and interpretation of data in this manuscript. All authors agree to be accountable for all aspects of work ensuring integrity and accuracy. All authors contributed to the article and approved the submitted version.

## Funding

This work was supported by the National Natural Science Foundation of China (Nos. 82160282, 82160768, and 82002822) and Natural Science Foundation of Guangxi Province (No. GuikeZD20302006).

## Conflict of Interest

The authors declare that the research was conducted in the absence of any commercial or financial relationships that could be construed as a potential conflict of interest.

## Publisher's Note

All claims expressed in this article are solely those of the authors and do not necessarily represent those of their affiliated organizations, or those of the publisher, the editors and the reviewers. Any product that may be evaluated in this article, or claim that may be made by its manufacturer, is not guaranteed or endorsed by the publisher.

## Abbreviations

LUAD: lung adenocarcinoma
COVID-19: coronavirus disease 2019
TCGA: The Cancer Genome Atlas
GO: Gene Ontology
BP: Biological process
KEGG: Kyoto Encyclopedia of Genes and Genomes
IPA: Ingenuity Pathway Analysis.
